# Effect of vitamin D supplementation on health status in non-vitamin D deficient people with type 2 diabetes mellitus

**DOI:** 10.1530/EC-16-0070

**Published:** 2016-11-16

**Authors:** S Westra, Y H M Krul-Poel, H J van Wijland, M M ter Wee, F Stam, P Lips, F Pouwer, S Simsek

**Affiliations:** 1 Department of Internal MedicineMedical Centre Alkmaar, Alkmaar, the Netherlands; 2 Department of General PracticeDIAZON, Alkmaar, the Netherlands; 3 Department of Epidemiology and BiostatisticsVU Medical Centre, Amsterdam, the Netherlands; 4 Department of Internal MedicineEndocrine Section, VU Medical Centre, Amsterdam, the Netherlands; 5 Department of Medical and Clinical PsychologyTilburg University, Tilburg, the Netherlands

**Keywords:** RCT, vitamin D, health-related quality of life, type 2 diabetes mellitus

## Abstract

**Objective:**

Increased levels of depressive symptoms, fatigue or pain (all dimensions of reduced health-related quality of life (HRQOL)) are common in people with type 2 diabetes mellitus (DM). Earlier studies have reported associations between low vitamin D status and fatigue and depressive symptoms. The aim of the present study was to examine the effects of vitamin D supplementation on dimensions of HRQOL in people with type 2 DM.

**Design:**

Randomised, double-blind, placebo-controlled trial.

**Methods:**

The effect of monthly cholecalciferol 50,000 IU vs placebo on HRQOL was assessed in 275 adults with type 2 DM derived from general practices. HRQOL at baseline and after six months using the Short Form 36 Health Survey (SF-36) was collected. Linear regression analyses were used to compare the change in HRQOL over time between the vitamin D and placebo group.

**Results:**

187/275 (68%) completed baseline and follow-up SF-36 and were included in the analysis. Median serum 25-hydroxyvitamin D almost doubled in the intervention group compared to that in the placebo group (58.5–106.0 nmol/L vs 60.0–61.5 nmol/L, respectively). A small significant difference (adjusted B: −8.90; 95% CI: −17.16 to −0.65) between both groups was seen concerning the SF-36 domain role limitations due to physical problems in disadvantage of the vitamin D group.

**Conclusions:**

Six months of vitamin D supplementation did not improve HRQOL in non-vitamin D-deficient people with type 2 DM managed on oral antidiabetic therapy.

## Introduction

With a total number of 415 million people in 2015, expecting to increase to a number of 642 million people in 2040, diabetes mellitus (DM) is a growing worldwide epidemic. It is common knowledge that people with DM are at increased risk for microvascular and macrovascular complications, including neuropathy, nephropathy, retinopathy, peripheral artery disease and cardiovascular disease (1). Furthermore, in people with type 2 diabetes mellitus (type 2 DM), relatively high prevalences of depression, fatigue and (neuropathic) pain were found resulting in a decreased quality of life (2, 3, 4, 5, 6). Depressive symptoms and fatigue in people with diabetes are related to an increased risk of developing diabetes-specific complications (2, 3). Moreover, people with depressive symptoms and diabetes had an almost 50% increased all-cause mortality rate, probably due to non-optimal self-care (2).

Low vitamin D status is common in people with type 2 DM (7), and previous observational studies demonstrated an association between low vitamin D status and a reduced health-related quality of life (HRQOL), fatigue and depressive symptoms (8, 9, 10, 11, 12, 13, 14). Two recent meta-analyses (15, 16) based on the results of randomised controlled trials, which investigated the effect of vitamin D supplementation on depressive symptoms, suggest an improvement of depressive symptoms after vitamin D supplementation (15, 16). Intervention studies concerning the effect of vitamin D supplementation on fatigue are scarce, and the studies that have been executed are difficult to compare as their research designs are very different. Lima and coworkers (17) performed a randomised placebo-controlled trial in adolescents and young adults with juvenile systemic lupus erythematosus and found a significant reduction of ‘fatigue-related to social life’ score (when using the Kids Fatigue Severity Scale) in the vitamin D group compared to placebo after 24 weeks of oral cholecalciferol 50,000 IU per week (17). In addition, a significant improvement in fatigue score in all five scales (general, physical, emotional, mental and vigour) of the Multidimensional Fatigue Symptom Inventory Short Form was seen in primary care people with a low vitamin D status and fatigue as their main problem, after five weeks of vitamin D supplementation (oral ergocalciferol 50,000 IU three times per week) (18). However, this study was not blinded or placebo-controlled. It should be noted that the majority of the people included in these studies did not have type 2 DM.

The biological mechanisms linking vitamin D status to HRQOL, depressive symptoms and fatigue in people with type 2 DM are not clear. Hypothetically, vitamin D deficiency may contribute to poor glycaemic control (19), which in turn leads to a higher risk to develop microvascular and macrovascular complications in the long term (19). Furthermore, due to the immunomodulatory properties of vitamin D and its association with oxidative stress, vitamin D may influence low-grade systemic inflammation, which is linked to both depressive symptoms and insulin resistance (20, 21). Another possible link between vitamin D status and depressive symptoms is an elevated parathyroid hormone (PTH) level that has been linked to depressive symptoms and insulin resistance and is increased in the state of vitamin D deficiency (22, 23). Moreover, vitamin D itself seems to have cardioprotective effects as well (24). Based on these findings, we hypothesise a positive effect of vitamin D supplementation on fatigue and depressive symptoms in people with type 2 DM. The aim of this study was to test whether six months of vitamin D supplementation improves the Short Form 36 (SF-36) Health Survey domain scores, especially the domains’ physical functioning, role limitations due to physical problems, social functioning, role limitations due to emotional problems, mental health and vitality, in people with type 2 DM, using a randomised double-blind placebo-controlled trial design.

## Subjects and methods

### Study design and patients

The SUNNY trial (acronym for StUdy the effect of vitamiN D supplemeNtation on glYcaemic control in type 2 DM) is a double-blind randomised placebo-controlled clinical trial, with the primary aim to determine the effect of vitamin D supplementation on glycaemic control in people with type 2 DM (25). Secondary aim was to investigate whether vitamin D supplementation improved the dimensions of HRQOL (25). The trial was conducted in five general practices in and around the city of Alkmaar, the Netherlands, latitude 52°, between July 2012 and April 2013. Adult persons (≥18 years) with type 2 DM treated with lifestyle advice, metformin, and/or sulfonylurea derivatives (SU derivatives) were invited for participation in the study. Serum HbA_1c_ had to be stable and below or equal to 8.0% (64 mmol/mol) for the last three months without recent changes in hypoglycaemic agents. Main exclusion criteria were an impaired renal function (estimated glomerular filtration rate (eGFR) <30 mL/min calculated from serum creatinine using the MDRD formula), any granuloma forming disorder, hypercalcaemia (serum calcium >2.65 nmol/L) of any reason, serum 25-hydroxyvitamin D (25(OH)D) <15 nmol/L or >150 nmol/L, urolithiasis, psychiatric treatment for schizophrenia, organic mental disorder or bipolar disorder currently or in the past, insufficient knowledge of the Dutch language and substance abuse (other than nicotine) or no signed informed consent. Withdrawal criteria for premature termination of the trial were increase of HbA_1c_ 69 mmol/mol (>8.5%), hypersensitivity to cholecalciferol or placebo, onset of urolithiasis, any change in antidiabetic medication or serum 25(OH)D <15 or >250 nmol/L, and during the study, people were not allowed to take vitamin D supplements.

This trial protocol was approved by the Medical Ethics Committee of Noord-Holland, the Netherlands and was conducted according to the principles of the Declaration of Helsinki (NTR3154). A detailed description of the protocol can be found elsewhere (25). Consent of all participants was obtained after full explanation of the purposes and nature of all procedures used in the SUNNY trial.

### Intervention

All participants were randomised according to either an oral dose of cholecalciferol 50,000 IU or an identically looking placebo once a month for 6 months (Meander Medical Center, Amersfoort, the Netherlands).

### Outcome measures

Change in HRQOL after six months of vitamin D supplementation was one of the secondary outcomes described in the study protocol of the SUNNY trial (25). HRQOL was assessed at baseline and six months after baseline, using the Dutch version of the Short Form 36 (SF-36) Health Survey, which was translated and validated by Aaronson and coworkers in 1994 (26). The SF-36 questionnaire is composed of 36 questions and represents eight domains and two summary measures: physical functioning, role limitations due to physical problems, bodily pain, general health perceptions (together presenting the physical component summary), mental health, vitality, social functioning and role limitations due to emotional problems (together presenting the mental component summary). For each domain, scores are summed and converted to a scale from 0 to 100, with lower scores indicating a poorer HRQOL (27).

Demographic data, medical history, the use of vitamin D supplements and diabetes-specific elements (treatment, complications and duration) were collected from medical records and during interviews. Lifestyle information including smoking status (yes/no), alcohol use (units per week), sun exposure (hours per week) and physical activity (hours per week) were self-reported and gathered through interviews. Standard anthropometric data (height and weight) and venous blood collection were obtained from each person. Serum 25(OH)D was measured using an iSYS automated immunoanalyzer (IDS GmbH, Frankfurt, Germany). Data were collected at baseline and after six months.

### Randomisation

The participants were randomised 1:1 according to the method of block randomisation with a block size of 10. No stratification was used. The randomisation procedure was performed by the pharmacist. The participants and the research team remained blinded until the end of the study.

### Statistical analysis

People who completed the study (returned questionnaires at baseline and 6 months) were included in the statistical analyses. In case of one or two missing SF-36 domains, linear imputation was used. When more SF-36 domains were missing, the people were excluded. All data were analysed using the Statistical Package of the Social Sciences (SPSS software, version 20.0; SPSS Inc.). Baseline characteristics were presented as means ± s.d., frequencies (%) or as median (interquartile range (IQR)) in case of a skewed distribution.

Linear regression analysis was used to assess the mean difference between intervention and placebo group after six months (mean difference reported as *B* and *β*). Change in SF-36 domain score was analysed as a dependent outcome with randomisation group as an explanatory variable. To correct for regression to the mean, all analyses were adjusted for its baseline value. In case of skewed distribution, the separate SF-36 domains were log transformed.

As we know that men and women provide different outcome on the SF-36 questionnaire and oestrogen use may increase the concentration of the vitamin D-binding protein and improve hydroxylation of vitamin D in the liver, the models will be tested for effect modification by gender (26). Furthermore, all analyses were corrected for confounding variables, which were selected based on literature, including age, gender (if no effect modification), BMI and season of blood collection. Subgroup analyses were performed in people with low vitamin D status, defined as 25(OH)D <50 nmol/L according to the practical guidelines of the Endocrine Society and the Institute of Medicine.

A two-sided *P* value of <0.05 was considered as significant.

## Results

**Figure 1:**
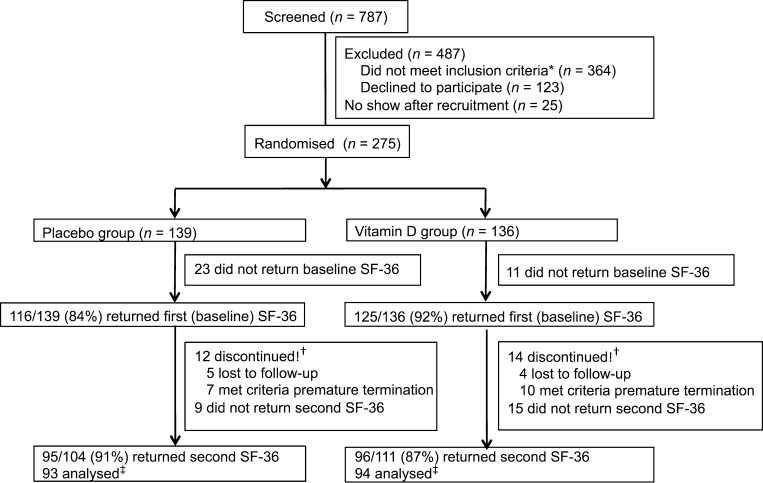
Participant flowchart. *Most people did not meet the inclusion criteria because of insulin therapy. ^†^Did not received second SF-36. ^‡^2 people excluded from analyses because ≥2 SF-36 domains were missing at baseline or follow-up.

A total number of 787 people were screened for eligibility of which 300 persons were recruited and finally 275 persons (no show: *n* = 25) were randomised to either vitamin D supplementation (*n* = 136) or placebo (*n* = 139) (Fig. 1). 487 (62%) people were excluded from the study because they did not meet the inclusion criteria (75%, mostly because they used insulin) or refused to participate (25%). During the trial, 17 people met the withdrawal criteria for premature termination due to change in antidiabetic medication (*n* = 10), HbA_1c_ >69 mmol/mol (>8.5%) (*n* = 5) or serum 25(OH)D <15 or >250 nmol/L (*n* = 2) and nine people were lost to follow-up. SF-36 response rate at baseline was 88% (241/275) and 89% at six months of follow-up (191/215), total SF-36 response rate was 70% (191/275). Linear imputation was executed in four people at baseline and two people at follow-up for the SF-36 domains role limitations due to physical problems, general health perceptions and role limitations due to emotional problems. Four people were excluded because information on two or more SF-36 domains were missing, resulting in 187/275 (68%) people with complete data.

Baseline demographic, clinical characteristics and HRQOL of all people included in the vitamin D group and in the placebo group are presented in Table 1. Mean age was 68 years ± 8 and 67% of the people were men. The median diabetes duration was 6 years (3–8) with a median HbA_1c_ of 51 (46–54 mmol/mol) (6.8 (6.4–7.1%)). Overall mean serum 25(OH)D was 61.1 ± 22.6 nmol/L. At baseline, 63 people (34%) had a serum 25(OH)D level of 50 nmol/L or less; a serum 25(OH)D level between 50 and 75 nmol/L was present in 79 people (42%) and 45 people (24%) had a serum 25(OH)D >75 nmol/L. After six months of vitamin D supplementation, the median 25(OH)D level almost doubled in the vitamin D group from 58.5 (43.0–75.0) to 106.0 (85.0–117.0) nmol/L, whereas in the placebo group, the 25(OH)D level remained stable (serum 25(OH)D: 60.0 (44.0–74.0) to 61.5 (37.0–85.5) nmol/L). In the intervention group, 73% of the people achieved a serum 25(OH)D level ≥75 nmol/L at three months, and 84% after six months of vitamin D supplementation. No differences in baseline characteristics were seen between the people who were randomised (*n* = 275) and those finally analysed (*n* = 187) (data not shown).

**Table 1:** Baseline demographic and clinical characteristics in the vitamin D group and the placebo group (*n* = 187).

	**Vitamin D group** (*n* = 94)	**Placebo group** (*n* = 93)
Demographic parameters		
Age (years)	67 ± 8	68 ± 9
Male	68 (72)	57 (61)
Diabetes duration (years)	6 (3–8)	6 (4–8)
White skin colour	91 (95)	90 (95)
Antidiabetic treatment		
Lifestyle adjustments	3 (3)	6 (7)
Metformin	66 (70)	48 (52)
SU derivatives	2 (2)	5 (5)
Metformin and SU derivatives	23 (25)	34 (37)
Microvascular complications* ≥1	25 (27)	13 (14)
Cardiovascular disease, yes	28 (30)	33 (36)
Single	8 (9)	20 (22)
Education level		
Low	63 (69)	64 (72)
Middle	21 (23)	18 (20)
High	7 (8)	8 (9)
Employment status		
Paid employment	24 (26)	25 (26)
Unemployed or incapacitated	8 (9)	7 (8)
Retired	62 (66)	61 (66)
Alcohol use >2 units/day	12 (13)	12 (13)
Current smoker	15 (16)	13 (14)
Use of vitamin D supplements^†^	14 (15)	9 (10)
Physical activity		
<2 h/week	31 (33)	22 (24)
2–5 h/week	40 (43)	52 (56)
>5 h/week	23 (25)	19 (20)
Sun exposure (%)		
<5 h/week	34 (36)	37 (40)
5–10 h/week	46 (49)	44 (47)
>10 h/week	14 (15)	12 (13)
Season of blood collection		
Spring	12 (13)	8 (9)
Summer	23 (25)	20 (22)
Autumn	43 (46)	49 (53)
Winter	16 (17)	16 (17)
Clinical characteristics		
BMI (kg/m^2^)	27.7 (26.0–31.2)	27.5 (25.3–30.6)
HbA_1c_ (mmol/mol)	51 (46–55)	51 (46–53)
HbA_1c_ (%)	6.8 (6.4–7.2)	6.8 (6.4–7.0)
Serum 25(OH)D (nmol/L)	59.0 (43.0–75.0)	60.0 (44.0–74.0)
Serum PTH (pmol/L)	5.1 (3.8–6.8)	5.2 (4.0–6.5)
Health-related quality of life		
Physical functioning	85 (70–95)	85 (65–95)
Role limitations physical	100 (50–100)	100 (50–100)
Bodily pain	74 (52–100)	74 (62–100)
General health perceptions	67 (47–77)	62 (47–72)
Mental health	88 (76–92)	80 (64–92)
Role limitations emotional	100 (100–100)	100 (100–100)
Vitality	75 (60–85)	70 (55–85)
Social functioning	100 (88–100)	100 (75–100)
Physical component summary	80 (60–91)	76 (63–87)
Mental component summary	87 (74–91)	82 (70–90)

Continuous variables are presented in mean ± s.d. or median (IQR) depending on normality. Categorical variables are presented in numbers (%).

* Including retinopathy, nephropathy and neuropathy. ^†^Maximum dose of 400 IU vitamin D supplement daily before the start of the trial.

25(OH)D, 25 hydroxyvitamin D; PTH, parathyroid hormone; SU derivatives, sulphonylurea derivatives.

### Serum 25(OH)D and HRQOL

**Figure 2:**
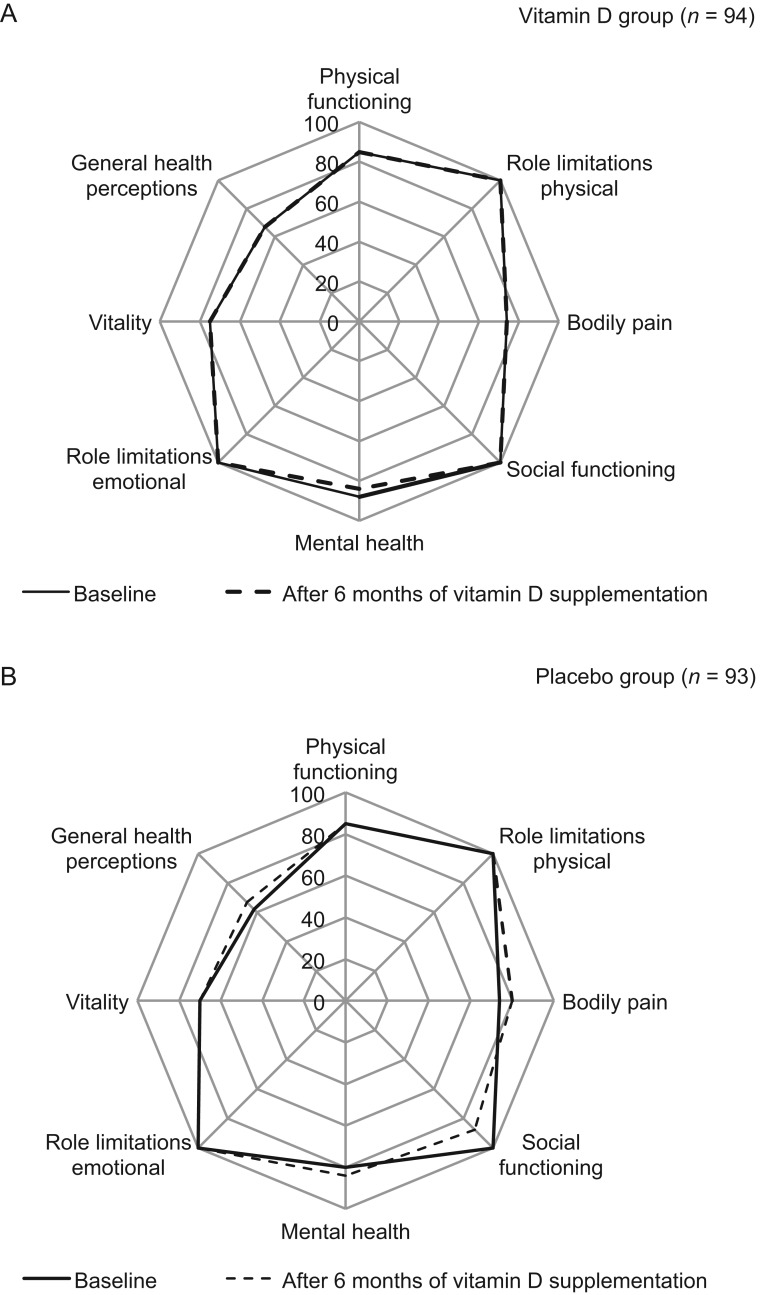
Health-related quality of life domains (SF-36) in the vitamin D group (A) and placebo group (B); baseline vs after six months of vitamin D supplementation (*n* = 187).

The present study revealed that vitamin D supplementation did not affect HRQOL (Fig. 2 and Table 2) in people with type 2 DM. No effect modification by gender was seen (data not shown). A small significant difference, to the detriment of the vitamin D group, was observed in the SF-36 domain role limitations due to physical problems (adjusted B: −8.90; 95% CI: −17.16 to −0.65).

**Table 2:** Health-related quality of life (SF-36 domains) in the vitamin D group and the placebo group (*n* = 187).

	**Δ Vitamin D group** (*n* = 94)	**Δ Placebo group** (*n* = 93)	β*	B*	**95% CI**	***P***
Physical functioning	−0.55 ± 12.77	1.21 ± 11.70	−0.062	−1.51	−4.99; 1.96	0.39
Role limitations physical	−5.32 ± 32.77	4.84 ± 32.61	−0.138	−8.9	−17.16; −0.65	0.04^†^
Bodily pain	−0.24 ± 19.33	2.40 ± 16.59	−0.07	−2.52	−7.30; 2.27	0.3
General health perceptions	0.37 ± 13.39	3.10 ± 13.61	−0.063	−1.71	−5.44; 2.02	0.37
Mental health	−1.68 ± 11.78	−0.12 ± 13.09	−0.033	−0.83	−4.42; 2.77	0.65
Role limitations emotional	−3.72 ± 34.92	1.08 ± 33.50	−0.063	−4.31	−13.00; 4.37	0.31
Vitality	−2.71 ± 13.35	−1.00 ± 12.17	−0.064	−1.62	−5.11; 1.88	0.36
Social functioning^‡^	0.00 (−12.50 to 0.00)	0.00 (−12.50 to 0.00)	0.95	0.95	0.80; 1.11	0.49
Physical component summary	−1.50 ± 13.82	2.89 ± 11.39	−0.15	−3.77	−7.26; −0.28	0.04^†^
Mental component summary^‡^	0.79 (−6.38 to 6.00)	0.00 (−4.50 to 7.50)	0.93	0.97	0.91; 1.04	0.34

A positive *β* value indicates an increase in the SF-36 domain in the vitamin D group compared to the placebo group.

* Adjusted for age, gender, BMI, baseline SF-36 domain, baseline 25-hydroxyvitamin and season of blood collection; ^†^*P* < 0.05; ^‡^Using log-transformed values; *β* of 0.95 (social functioning) indicating a 5% lower SF-36 score in the vitamin D group vs the placebo group after six months.

In the group people with 25(OH)D <50 nmol/L (34%), mean age was 67 years ± 8, 56% of the people were men and mean serum 25(OH)D was 38 ± 8 nmol/L. Linear regression revealed no differences in HRQOL between the vitamin D and placebo group in this pre-specified subgroup analysis (data not shown).

## Discussion

In this randomised, double-blind, placebo-controlled trial in Dutch people with well-controlled type 2 DM treated in general practice, we found a statistically significant decline (B: −8.90; 95% CI: −17.16 to −0.65) in the SF-36 domain ‘role limitations due to physical problems’ after six months of vitamin D supplementation. However, concerning the remaining SF-36 domains, no effect of vitamin D supplementation was found.

Before interpreting the results of our study, it should be emphasised that the SF-36 domain scores were not standardised, and they are calculated from different numbers of questions with different types of set response choices resulting in a fixed value per question, which is domain specific. Considering the SF-36 domain role limitations due to physical problems, which represent only four yes or no questions, thus valuing every question with twenty-five points, we interpret the statistically significant finding with a beta of only 0.138 (B: −8.90; 95% CI: −17.16 to −0.65) as clinically not relevant (27).

Other studies exploring the effect of vitamin D on HRQOL in people with diabetes are scarce. A recent systematic review from Hoffmann and coworkers (28), categorised fifteen articles (of which seven randomised placebo-controlled trials), which examined the effect of vitamin D supplementation on HRQOL according to length of intervention (more or less than six months) and study population (healthy vs diseased people; no studies focusing on diabetes were included). In contrast to our results, in four of the seven studies, which were derived from the group with diseased people and vitamin D intervention for six months or less, an improvement of HRQOL (especially in the domains role limitations due to physical problems, bodily pain, vitality and physical functioning; however, only two studies used (a variation of) the SF-36) after vitamin D supplementation was found, which was interpreted by the investigators as evidence for an small-to-moderate positive effect of short-term vitamin D supplementation on HRQOL in diseased people (28). However, no meta-analysis could be done due to the great heterogeneity in study samples, dose and type of vitamin D supplementation and the variation of HRQOL instruments that had been used. The before-mentioned study results should be viewed with caution as the quality of evidence is low due to poor methodological quality. Also, many of the differences in HRQOL that have been reported were small and not likely to be of value in the clinical setting. In addition, the only randomised placebo-controlled trial in this review with the maximal points for methodology, found no effect of vitamin D supplementation (daily oral 800 IU vitamin D3) on the physical component summary or mental component summary in elderly people >70 years with previous low trauma osteoporotic fracture using the SF-12 (shortened version of the SF-36) after 24–62 months of follow-up (29).

Moreover, one recent double-blind, placebo-controlled study including 60 people receiving haemo­dialysis of whom 55% had a history of diabetes, did not demonstrate an effect of vitamin D supplementation (cholecalciferol 50,000 IU/week for eight weeks followed by 50,000 IU/month for four months) on HRQOL (using KDQOL-36, a kidney disease-specific measure of HRQOL including several parts of the SF-36 questionnaire) after six months of follow-up (30).

The main limitation of our study, which could explain that we found no positive effect of vitamin D supplementation on HRQOL in the present study, is the relatively good baseline HRQOL of several SF-36 domains in our study population that may have resulted in ceiling effects. In addition, the SF-36 domain scores in our study population are comparable with the SF-36 domain scores in the general Dutch population (26), suggesting low disease burden with few mental and physical limitations, leaving almost no opportunity for improvement. The low disease burden in our study population is also reflected in the small number of people with one or more than one microvascular complications (*n* = 38, 20%) and the good glycaemic control with a median HbA_1c_ of 51 (46–54) mmol/mol (6.8 (6.4–7.1%)).

Furthermore, when expecting a positive effect of vitamin D supplementation on HRQOL by reducing systemic low-grade inflammation or improving glycaemic control leading to reduced or less severe diabetes-specific complications, the relatively short duration of the trial could be another reason for not finding an improvement of HRQOL after vitamin D supplementation.

Last, with a median 25(OH)D level of 58.5 nmol/L (43.0–75.0) in the vitamin D group at baseline, our subjects are already replete in vitamin D according to the current guidelines of the Institute of Medicine from 2011, which defines vitamin D deficiency as 25(OH)D <50 nmol/L in respect to bone health. However, Spedding and coworkers (31) suggested different 25(OH)D levels required for non-skeletal diseases and reported a minimum 25(OH)D level of 75 nmol/L for reducing depressive symptoms (level II evidence: randomised controlled trial) (31). With a median 25(OH)D level of 106.0 (85.0–117.0) in the vitamin D group at the end of the study, vitamin D intervention was effective to increase 25(OH)D concentration to a level of which an improvement in depressive symptoms could be expected.

The strengths of our study are the randomised, double-blind, placebo-controlled design, the use of a well-validated questionnaire to determine HRQOL and the large study population.

In conclusion, six months of vitamin D supplementation did not improve HRQOL in people with tightly controlled type 2 DM derived from general practices. Longitudinal studies in people with poorly controlled type 2 DM, with multiple measurements over time concerning physical limitations, mental health and vitality and factors possible affecting these domains including low 25(OH)D level, inflammation factors, diabetes-specific treatment and complications and lifestyle factors are necessary to understand and eventually affect, the relationship between diabetes and a reduced (health-related) quality of life.

## Declaration of interest

The authors declare that there is no conflict of interest that could be perceived as prejudicing the impartiality of the research reported.

## Funding

This research did not receive any specific grant from any funding agency in the public, commercial or not-for-profit sector.

## Author contribution statement

S S and Y K initiated the study. Y K and S W performed the data collection and statistical analyses together with M t W. S W wrote the manuscript and Y K edited the manuscript. S S first revised the paper critically. Thereafter, F S, H W, F P, M t W and P L revised the paper critically. S S is the guarantor of this work and, as such, had full access to all the data in the study and takes responsibility for the integrity of the data and the accuracy of the data analyses. All the authors were involved in the final approval of the version to be published.
